# Poorer dynamic postural stability in patients with anterior cruciate ligament rupture combined with lateral meniscus tear than in those with medial meniscus tear

**DOI:** 10.1186/s43019-019-0027-x

**Published:** 2020-01-01

**Authors:** Jin Hyuck Lee, Dae-Hee Lee, Jong-Hoon Park, Dong Won Suh, Eunseon Kim, Ki-Mo Jang

**Affiliations:** 1 Department of Sports Medical Center, , Anam Hospital, Korea University College of Medicine, Seoul, South Korea; 2 Department of Orthopaedic Surgery, Samsung Medical Center, Sungkyunkwan University School of Medicine, Seoul, South Korea; 3 Department of Orthopaedic Surgery, Anam Hospital, Korea University College of Medicine, 73 Inchon-ro (Anam-dong 5-ga) Seongbuk-gu, Seoul, 02841 South Korea; 4 Department of Orthopaedic Surgery, Barunsesang Hospital, Seongnam, South Korea

**Keywords:** Anterior cruciate ligament tear, Meniscus tear, Postural stability, Knee muscle strength

## Abstract

**Background:**

Only limited data are available regarding postural stability between anterior cruciate ligament (ACL)-injured patients with medial meniscus (MM) tear and those with lateral meniscus (LM) tear. The purpose of this study was to compare preoperative postural stability for both involved and uninvolved knees in ACL rupture combined with MM and LM tears. It was hypothesized that there would be a significant difference in postural stability between these two groups.

**Methods:**

Ninety-three ACL-injured patients (53 combined with MM tears vs. 40 combined with LM tears) were included. Static and dynamic postural stability were evaluated with the overall stability index (OSI), anterior–posterior stability index (APSI), and medial–lateral stability index (MLSI) using stabilometry. Knee muscle strength was evaluated using an isokinetic testing device.

**Results:**

In the static postural stability test, none of the stability indices showed significant differences between the two groups for both knees (*p* > 0.05). In the dynamic postural stability test for involved side knees, the OSI and APSI were significantly higher in the LM tear group compared to the MM tear group (OSI: 2.0 ± 0.8 vs. 1.6 ± 0.5, *p* = 0.001; APSI: 1.5 ± 0.6 vs. 1.3 ± 0.5, *p* = 0.023), but not the MLSI (*p* > 0.05). In the static and dynamic postural stability tests in each group, there were no significant differences between the involved and uninvolved side knees (*p* > 0.05). There was no significant difference in the knee muscle strength between the two groups (*p* > 0.05). All postural stability showed no significant correlation with knee muscle strength (*p* > 0.05).

**Conclusion:**

Dynamic postural stability was poorer in patients with ACL rupture combined with LM tear than in those with MM tear. Therefore, close monitoring for postural stability would be necessary during preoperative and postoperative rehabilitation, especially for patients with ACL rupture combined with LM tear.

**Level of evidence: Level III:**

## Introduction

The anterior cruciate ligament (ACL) is one of the most frequently injured structures in the knee joint, particularly in young and active patients. ACL injuries are commonly accompanied with injury in one or both menisci [1, 2]. The incidence of accompanied meniscal tear varies considerably, ranging from 16 to 82% in acute ACL injuries and up to 96% in chronic ACL insufficiency [3, 4]. The reported injury incidence is higher in the lateral meniscus (LM) in acute ACL injuries, whereas the medial meniscus (MM) is more frequently injured in chronic ACL insufficiency [1, 5].

The ACL and menisci play an important role in biomechanical functions of the knee joint. They contain some mechanoreceptors that affect proprioception and neuromuscular control [6–8]. A recent meta-analysis reported that patients with ACL or meniscus injuries have impaired proprioception, due to the loss of both slow-adapting (Ruffini endings) and rapid-adapting (Pacinian corpuscles) mechanoreceptors [9, 10]. Therefore, previous studies have demonstrated that there is impaired postural stability in patients with ACL or meniscus injuries [11–14].

It was reported that some differences exist in the distribution of mechanoreceptors between the LM and the MM [15, 16]. In addition, in a previous biomechanical study, Peña et al. [17] reported that axial femoral compressive loads and maximal shear stress increased by 200% more after lateral meniscectomy than after medial meniscectomy; thus, LM tear may increase joint instability more than MM tear, resulting in decreased postural stability. Therefore, it is expected that postural stability might differ between patients with MM and LM injuries. However, there is a lack of studies that establish a comparison of postural stability in patients with MM and LM tears. A recent study has demonstrated a significant difference in postural stability between MM and LM tears [18]. However, to our knowledge, no study has managed to directly compare postural stability in patients with ACL injury accompanied by MM and LM tears (ACL rupture combined with MM tear vs. ACL rupture combined with LM tear).

Impaired postural stability may result in impaired knee joint function and increased risk of future injuries [11, 19]. Identifying differences in preoperative postural stability between ACL-injured patients with MM tear and those with LM tear might help us to optimize the preoperative and postoperative rehabilitation protocols and reduce possible risks of future injuries. Therefore, the purpose of the present study is to compare preoperative postural stability in ACL rupture combined with MM tears or with LM tears. It was hypothesized that there would be a significant difference in postural stability between these two groups.

## Materials and methods

### Participants

This study complied with the Declaration of Helsinki and was approved by the institutional review board of our institute (IRB No.: 2017AN0178). Informed consent was obtained from all individual participants included in the study. This study retrospectively reviewed 195 patients who had undergone ACL reconstruction with meniscectomy or meniscus repair for ACL rupture with MM or LM tears in our institution from 2011 to 2017. Preoperative postural stability and muscle strength were assessed routinely on the day before surgery. We excluded patients with both meniscus tears in the same knee joint, discoid meniscus, revisional ACL reconstruction, prominent knee osteoarthritis (OA) signs on plain radiographs (Kellgren–Lawrence grade III or IV), a history of previous knee injury and operation, or meniscus tears in bilateral knees. Patients were also excluded if they are unable to perform the test devices (postural stability system or isokinetic muscle strength system) due to knee joint pain or limited range of motion, neuromuscular dysfunction, or visual impairment. Of the 195 patients in this retrospective case–control study, 93 subjects (53 ACL rupture with MM tear vs. 40 ACL rupture with LM tear) were finally enrolled in the current study. There was no significant difference in characteristics including age, sex, and BMI between the two groups (Table 1).

**Table 1:** Demographic data of enrolled patients

	ACL rupture with MM tear	ACL rupture with LM tear	*p* value
Sample size (*n*)	53	40	
Gender, male/female (*n*)	38/15	30/10	
Age (years)^a^	30.6 ± 6.9	29.3 ± 7.7	0.384
Height (cm)^a^	173.8 ± 6.5	172.6 ± 6.6	0.397
Weight (kg)^a^	68.2 ± 10.2	69.5 ± 8.8	0.538
Body mass index (kg/m^2^)^a^	22.5 ± 2.7	23.3 ± 3.2	0.167

*ACL* anterior cruciate ligament, *LM* lateral meniscus, *MM* medial meniscus

^a^Values expressed as the mean ± standard deviation

### Assessment of postural stability

**Fig. 1:**
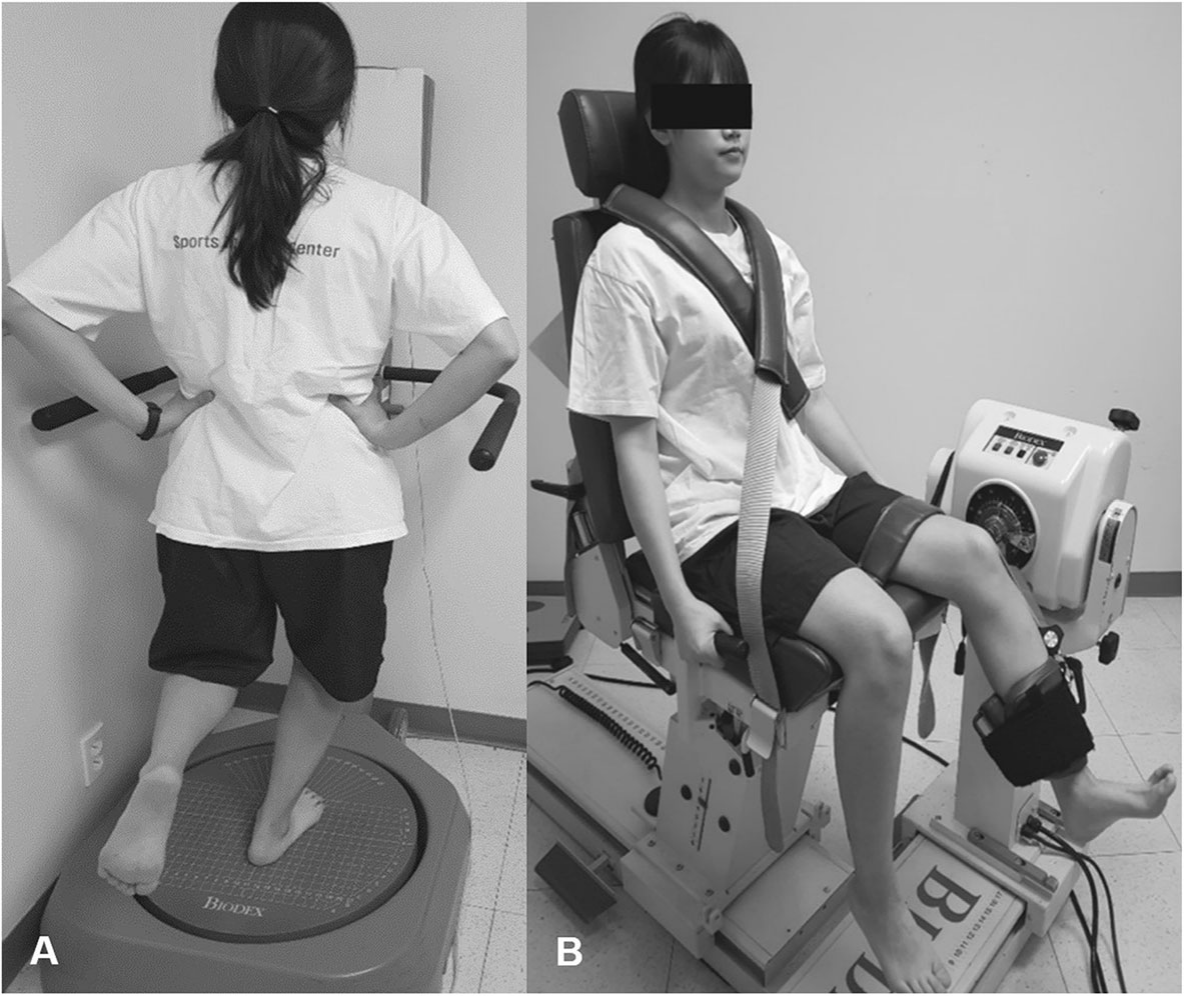
**a** Assessment of postural stability using the Biodex Stability System (BSS) (Biodex Medical Systems, Shirley, NY, USA). The static postural stability test instructed the subject to maintain the posture to level 12 platform (stable surface), whereas the dynamic postural stability test was measured on the same platform while decreasing platform stability gradually from level 12 (most stable) to level 1 (most unstable). **b** Assessment of isokinetic muscle strength using the Biodex multi-joint system 4 (Biodex Medical Systems). Each subject was seated on the device, with hips and knees flexed to 90° and trunk perpendicular to the floor. A strap was used to immobilize each subject’s thigh. The lateral femoral condyle of the knee joint was aligned with the rotational axis of the isokinetic machine

Postural stability was evaluated using the Biodex Stability System (BSS) (Biodex Medical Systems, Shirley, NY, USA). The foot platform surface of the BSS can move from 0° to 20° tilt in any direction. Each subject stood barefoot, and was instructed to stand with 90° flexion of the opposite knee on the platform, with their arms held at the pelvis (Fig. 1a). An examiner recorded the foot location of the lateral malleolus and the heel cord on the foot plate. The static single-leg balance test was instructed to maintain the posture to level 12 platform (stable surface). The dynamic single-leg balance test measured a change in posture for each level condition while decreasing platform stability gradually from level 12 (most stable) to level 1 (most unstable), with the stability level automatically declining every 1.66 s. If each individual was unable to maintain the balance until the end of the test, that test was terminated. Each test consisted of two trials performed for 20 s each for the two tests, with 10 s between each pair of tests. The mean and standard deviation of the two trials were calculated by the BSS for all postural stability parameters including overall stability index (OSI), anterior–posterior stability index (APSI), and medial–lateral stability index (MLSI) scores. A lower index score indicates good postural stability [18].

### Assessment of isokinetic muscle strength

Isokinetic knee muscle strength (concentric/concentric muscle contraction for extension/flexion) was measured with each subject seated on the Biodex multi-joint system 4 (Biodex Medical Systems), with hips and knees flexed to 90° and trunk perpendicular to the floor. A strap was used to immobilize each subject’s thigh. The lateral femoral condyle of the knee joint was aligned with the rotational axis of the isokinetic machine (Fig. 1b). Each test session consisted of five isokinetic knee flexions and extensions (range of motion, 90 to 0°) of each leg at 60°/s, with a rest time of 30 s between tests. Peak flexion and extension torques were recorded (Newton meter per kilogram). Flexor strength was regarded as hamstring muscle strength, while extensor strength was regarded as quadriceps muscle strength. The mean value of two trials was regarded as the maximal peak torque of the quadriceps and hamstring muscles.

### Statistical analysis

Based on a previous study for postural stability in patients with knee joint injuries [18, 20], an OSI difference > 0.5 between the ACL rupture combined with MM tear and combined with LM tear groups was considered significant. A power analysis was performed to determine the sample size, with a power of 0.8 and an α level of 0.05. A pilot study with five knees in each group indicated that 42 knees would be required to detect a significant difference. The power for detecting between-group differences for postural stability in this study was 0.804. Student’s *t* test was used to compare the differences for the static and dynamic postural stability and the knee muscle strength in the involved and uninvolved side knees between the two groups (ACL rupture combined with MM tear vs. ACL rupture combined with LM tear). A paired *t* test was used to compare all variables between the involved and uninvolved side knees in each group. The level of correlations between the static and dynamic postural stability and the knee muscle strength were assessed by Pearson’s coefficient of correlation (*r*) in each group. The level of statistical significance was set at *p* < 0.05. Data were analyzed using SPSS software version 17.0 (SPSS Inc., Chicago, IL, USA).

## Results

### Comparison of postural stability in involved side knees between the ACL-MM group and the ACL-LM group

In the comparison of the static postural stability test between the two groups, all three stability indices, the OSI, APSI, and MLSI, showed no significant difference in both involved and uninvolved side knees (*p* > 0.05). However, in the comparison of the dynamic postural stability test between the two groups, the ACL rupture combined with LM tear group indicated a significantly higher OSI and APSI in the involved side knees compared with the ACL rupture combined with MM tear group (OSI: 2.0 ± 0.8 vs. 1.6 ± 0.5, *p* = 0.001; APSI: 1.5 ± 0.6 vs. 1.3 ± 0.5, *p* = 0.023). However, there was no significant difference in the MLSI (1.0 ± 0.5 vs. 0.9 ± 0.4, *p* = 0.328) (Table 2).

**Table 2:** Comparison of the static and dynamic postural stability and the knee muscle strength in both knees between ACL rupture combined with MM tear and combined with LM tear groups

	Involved knee			Uninvolved knee		
	ACL rupture with MM tear	ACL rupture with LM tear	*p* value	ACL rupture with MM tear	ACL rupture with LM tear	*p* value
Static OSI	1.7 ± 1.0	1.8 ± 0.9	0.659	1.6 ± 0.7	1.6 ± 0.4	0.948
MD (95% CI)	− 0.1 (− 0.5, 0.3)			0 (− 0.2, 0.3)		
Static APSI	1.3 ± 0.9	1.4 ± 0.8	0.600	1.2 ± 0.6	1.3 ± 0.6	0.515
MD (95% CI)	− 0.1 (− 0., 0.3)			− 0.1 (− 0.3, 0.2)		
Static MLSI	1.2 ± 0.9	1.2 ± 0.8	0.852	1.1 ± 0.7	1.0 ± 0.5	0.513
MD (95% CI)	0 (− 0.3, 0.4)			0.1 (− 0.2, 0.3)		
Dynamic OSI	1.6 ± 0.5	2.0 ± 0.8	0.001^a^	1.7 ± 0.6	1.8 ± 0.6	0.619
MD (95% CI)	− 0.5 (− 0.8, − 0.2)			− 0.1 (− 0.3, 0.2)		
Dynamic APSI	1.3 ± 0.5	1.5 ± 0.6	0.023^a^	1.3 ± 0.5	1.3 ± 0.6	0.651
MD (95% CI)	−0.3 (− 0.5, 0)			0 (− 0.3, 0.2)		
Dynamic MLSI	0.9 ± 0.4	1.0 ± 0.5	0.328	1.0 ± 0.5	0.9 ± 0.4	0.154
MD (95% CI)	−0.1 (− 0.3, 0.1)			0.1 (0, 0.3)		
Quadriceps strength	109 ± 44.1	111 ± 42.4	0.819	200 ± 40.1	202 ± 38.9	0.811
MD (95% CI)	−2.0 (−20.1, 15.9)			−2.0 (−18.5, 14.5)		
Hamstring strength	49 ± 27.2	61 ± 31.6	0.078	100 ± 21.9	110 ± 27.2	0.062
MD (95% CI)	−12 (−23.1, 1.3)			−10 (− 19.7, 0.5)		

Values expressed as mean ± standard deviation

Measurement unit of postural stability and knee muscle strength was degrees and Newton meter per kilogram, respectively

*ACL* anterior cruciate ligament, *APSI* anterior–posterior stability index, *CI* confidence interval, *LM* lateral meniscus, *MD* mean difference, *MLSI* medial–lateral stability index, *MM* medial meniscus, *OSI* overall stability index

^a^Statistically significant

### Comparison of postural stability between involved and uninvolved side knees within groups

In the comparison of the static and dynamic postural stability tests, there was no significant difference in all three stability indices, the OSI, APSI, and MLSI, between the involved and uninvolved side knees in each group (*p* > 0.05) (Table 3).

**Table 3:** Comparison of the static and dynamic postural stability and the knee muscle strength between involved and uninvolved side knees in each group

	ACL rupture combined with MM tear			ACL rupture combined with LM tear		
	Involved knee	Uninvolved knee	*p* value	Involved knee	Uninvolved knee	*p* value
Static OSI	1.7 ± 1.0	1.6 ± 0.7	0.237	1.8 ± 0.9	1.6 ± 0.4	0.102
MD (95% CI)	−0.1 (− 0.3, 0.1)			− 0.2 (− 0.5, 0)		
Static APSI	1.3 ± 0.9	1.2 ± 0.6	0.226	1.4 ± 0.8	1.3 ± 0.6	0.250
MD (95% CI)	− 0.1 (− 0.3, 0.1)			−0.1 (− 0.4, 0.1)		
Static MLSI	1.2 ± 0.9	1.1 ± 0.7	0.385	1.2 ± 0.8	1.0 ± 0.5	0.217
MD (95% CI)	− 0.1 (− 0.3, 0.1)			− 0.2 (− 0.4, 0.1)		
Dynamic OSI	1.6 ± 0.5	1.7 ± 0.6	0.237	2.0 ± 0.8	1.8 ± 0.6	0.100
MD (95% CI)	0.1 (− 0.1, 0.3)			− 0.2 (− 0.6, 0.1)		
Dynamic APSI	1.3 ± 0.5	1.3 ± 0.5	0.827	1.5 ± 0.6	1.3 ± 0.6	0.166
MD (95% CI)	0 (− 0.2, 0.2)			−0.2 (− 0.5, 0.1)		
Dynamic MLSI	0.9 ± 0.4	1.0 ± 0.5	0.408	1.0 ± 0.5	0.9 ± 0.4	0.205
MD (95% CI)	0.1 (−0.1, 0.3)			−0.1 (− 0.4, 0.1)		
Quadriceps strength	109 ± 44.1	200 ± 40.1	< 0.001^a^	111 ± 42.4	202 ± 38.9	< 0.001^a^
MD (95% CI)	91 (78.3, 105.1)			91 (79.3, 103.9)		
Hamstring strength	49 ± 27.2	100 ± 21.9	< 0.001^a^	61 ± 31.6	110 ± 27.2	< 0.001^a^
MD (95% CI)	51 (42.9, 58.8)			49 (37.9, 61.2)		

Values expressed as mean ± standard deviation

Measurement unit of postural stability and knee muscle strength was degrees and Newton meter per kilogram, respectively

*ACL* anterior cruciate ligament, *APSI* anterior–posterior stability index, *CI* confidence interval, *LM* lateral meniscus, *MD* mean difference, *MLSI* medial–lateral stability index, *MM* medial meniscus, *OSI* overall stability index

^a^Statistically significant

### Comparison of the knee muscle strength test in involved and uninvolved side knees in the ACL-MM group and the ACL-LM group

The knee muscle strength was assessed using the maximal peak torque of the quadriceps and hamstring muscles. There was no significant difference in quadriceps and hamstring muscle strength in involved or uninvolved side knees between the two groups (*p* > 0.05) (Table 2). However, there were statistically significant decreases in quadriceps and hamstring muscle strength in the involved side knees compared with the uninvolved side knees in each group (*p* < 0.001) (Table 3).

### Correlation between the static and dynamic postural stability and the knee muscle strength

The results of correlation analysis between the static and dynamic postural stability and the knee muscle strength in the involved side knees in both the ACL rupture with MM tear and that with LM tear groups are presented in Table 4. There was no significant correlation between the static and dynamic postural stability and the knee muscle strength in each group (*p* > 0.05).

**Table 4:** Correlations between the static and dynamic postural stability and the knee muscle strength in the involved side knees in each group

		ACL rupture with MM tear		ACL rupture with LM tear	
		Quadriceps	Hamstring	Quadriceps	Hamstring
Static OSI	PCC (*r*)	−0.086	−0.141	0.136	0.228
	*p* value	0.541	0.313	0.403	0.157
Static APSI	PCC (*r*)	−0.104	−0.147	0.138	0.203
	*p* value	0.459	0.294	0.396	0.208
Static MLSI	PCC (*r*)	−0.101	−0.094	0.286	0.294
	*p* value	0.470	0.501	0.074	0.066
Dynamic OSI	PCC (*r*)	0.160	0.233	−0.097	−0.138
	*p* value	0.252	0.093	0.551	0.394
Dynamic APSI	PCC (*r*)	0.201	0.218	−0.004	−0.203
	*p* value	0.149	0.117	0.979	0.208
Dynamic MLSI	PCC (*r*)	0.028	0.219	−0.199	−0.001
	*p* value	0.841	0.115	0.218	0.996

*ACL* anterior cruciate ligament, *APSI* anterior–posterior stability index, *LM* lateral meniscus, *MLSI* medial–lateral stability index, *MM* medial meniscus, *OSI* overall stability index, *PCC* Pearson correlation coefficient

## Discussion

The current study compared the preoperative static and dynamic postural stability between ACL-injured patients with MM tear and those with LM tear. The most important finding of the present study was that dynamic postural instability was more prominent in the involved side knees in the ACL rupture with LM tear group than that in the ACL rupture with MM tear group. However, there was no significant difference in the uninvolved side knees. The static and dynamic postural stability was similar between the involved and uninvolved side knees in each group.

An injury to the ACL can compromise the neuromuscular function of the knee joint, resulting in impaired proprioception and dynamic stability of the knee joint [21–23]. Recent studies have suggested that the menisci also have important roles in neuromuscular control of the knee joint [1, 24, 25]. Therefore, previous studies have demonstrated that there is impaired postural stability in patients with ACL or meniscus injuries [11–14, 18]. However, to our knowledge, there has been no study directly comparing postural stability between ACL-injured patients with MM tear and those with LM tear. In the current study, we found that static postural stability showed no significant difference in both involved and uninvolved side knees between the two groups, whereas dynamic postural instability was more severe in the involved side knees of the ACL rupture combined with LM tear group compared with the ACL rupture with MM tear group.

Although the reasons for this result are unclear, one possible reason may be different anatomical features of the medial and lateral compartments of the knee joint. The opposing articular surfaces of the proximal tibia and distal femur in the lateral compartment articulate in a “convex on convex” manner, creating inherent instability in this region of the knee joint [26]. Although the medial compartment sustains higher load-bearing stresses, the LM covers a greater portion of the area in its compartment than does the MM [27, 28]. Moreover, the LM is potentially more movable to maintain its role in the compartment. Therefore, the LM tear can contribute more to postural instability than does the MM tear. Another possible reason is the different distribution and role of mechanoreceptors around the MM and the LM. O’Conner and McConnaughey [29–31] verified the existence of mechanoreceptors within the meniscus in animal studies. They demonstrated that Ruffini corpuscles (type I mechanoreceptor) were identified mainly in the posterior horn of the MM, whereas Pacinian corpuscles (type II mechanoreceptor) were mainly found in the posterior horn of the LM [31]. In addition, Day et al. [6] denoted that Pacinian corpuscles were not identified in the MM, but found only in the LM in human knees. Pacinian corpuscles respond rapidly to changes in dynamic joint motion while Ruffini corpuscles react slowly to changes in static joint position [32]. That is, static postural stability is more dependent on input of information from Ruffini corpuscles, whereas dynamic postural stability is more dependent on information from Pacinian corpuscles [33]. Therefore, more severe dynamic postural instability is expected in the LM tears than in the MM tears.

The results of the present study also showed that there were no significant differences in static and dynamic postural stability between the involved and uninvolved side knees in each group. The result of the current study might have originated from bilateral impairment of postural stability following a unilateral ACL or meniscus injury [20]. Previous studies have delineated that decreases of afferent neural signal input to the central nervous system after an injury of one limb resulted in loss of motor output in the opposite limb, thus leading to bilateral impairment [32, 34]. Park et al. [20] denoted that there was no significant difference in postural stability between the involved and uninvolved side knees in patients with ACL tear combined with meniscus tears, and the authors also suggested that bilateral impairment of postural stability is more severe in the ACL tear combined with meniscal tear group compared with the isolated ACL tear group. Therefore, we recommend that balance training should be emphasized during preoperative and postoperative rehabilitation programs for the uninvolved side knees as well as the involved side knees in patients with ACL rupture combined with meniscal tears. However, there are no normal values to justify bilateral impairment of postural stability in this study. Therefore, future studies which have normal values as a control would be necessary to confirm the bilateral impairment of postural stability more clearly.

In the comparison of thigh muscle strength, there were no significant differences in the quadriceps and hamstring muscles in either involved or uninvolved limbs between the two groups. However, there were statistically significant decreases in quadriceps and hamstring muscle strength in the involved side knees compared with the uninvolved side knees in both groups. Although there were no significant correlations between muscle strength and postural stability in the current study, previous studies demonstrated that the strength of knee muscles might affect postural stability [14, 35] In addition, in ACL-injured patients, deficits in knee muscle strength have been identified as an important negative predictor for both return to sports and self-reported function [36, 37]. Nonetheless, in patients with ACL rupture combined with meniscal tears, postoperative rehabilitation should be adjusted to protect meniscus repair, and could delay recovery of muscle strength. Consequently, close monitoring for muscle strengthening recovery would be necessary in patients with ACL rupture combined with meniscus tears rather than in isolated ACL injuries.

This study has several limitations. First, the study enrolled a relatively small number of patients in each group. However, we performed a power analysis to determine the sample size and enrolled more patients in comparison to the least necessary number. Second, we compared only preoperative conditions between the two groups. Future studies with postoperative serial change of postural stability would be necessary to demonstrate the differences more clearly between the two groups. Third, there might have been a visual compensation during the single-leg balance test, which could affect the results of postural stability test [38]. However, we reduced the possibility of biases in the postural stability test by covering the control screen of the dynamometer. Fourth, the patterns and extents of meniscus tears were not verified in each group. Previous studies reported that different distribution of mechanoreceptors, which might affect postural stability, was identified according to tear size and position in the medial and lateral meniscus [6, 39]. Therefore, further studies with subgroup analysis for patterns and extents of meniscus tears would be necessary to elucidate the results of the present study more clearly. Lastly, there is a lack of a control group composed of healthy subjects. We used data from the uninvolved side limbs as a control in each group. A control group of healthy subjects would make our results more meaningful.

## Conclusion

Dynamic postural stability was poorer in patients with ACL rupture combined with LM tear than in those with MM tear. Therefore, clinicians and physical therapists should take the results of this study into account in the management of ACL-injured patients with meniscus tears, and close monitoring for postural stability would be necessary during preoperative and postoperative rehabilitation, especially for patients with ACL rupture combined with LM tear.

## Data Availability

The data related to the current study are available from the corresponding author on reasonable request.

## Abbreviations

ACL: Anterior cruciate ligament
APSI: Anterior–posterior stability index
BSS: Biodex Stability System
LM: Lateral meniscus
MLSI: Medial–lateral stability index
MM: Medial meniscus
OA: Osteoarthritis
OSI: Overall stability index
